# Synchronous multimode ultrasound for assessing right-to-left shunt: a prospective clinical study

**DOI:** 10.3389/fneur.2023.1148846

**Published:** 2023-06-20

**Authors:** Qingyang Yao, Huahua Xiong, Daxue Zhang, Shuqun Ren, Wenwei Qi, Xia Zou, Yingying Zhao, Shanshan Huang, Jing Wang, Liming Cao

**Affiliations:** ^1^Department of Neurology, The First Hospital of Quanzhou Affliated to Fujian Medical University, Quanzhou, Fujian, China; ^2^Department of Ultrasound, Shenzhen Second People’s Hospital, Shenzhen, China; ^3^Department of Ultrasound, The First Affiliated Hospital of Shenzhen University, Shenzhen, China; ^4^School of Nursing, Anhui Medical University, Hefei, China; ^5^School of Nursing, Guangxi University of Chinese Medicine, Nanning, China; ^6^Tianjin Institute of Cardiology, The Second Hospital of Tianjin Medical University, Tianjin, China; ^7^Department of Neurology, The First Affiliated Hospital of Shenzhen University, Shenzhen, China; ^8^College of Pharmacy, Changsha Medical University, Changsha, China

**Keywords:** synchronous test, multimode ultrasound, contrast-transthoracic echocardiography, contrast-transcranial Doppler, right-to-left shunt, test effect

## Abstract

**Background:**

Right-to-left shunt (RLS) is associated with several conditions and causes morbidity. In this study, we aimed to evaluate the effectiveness of synchronous multimode ultrasonography in detecting RLS.

**Methods:**

We prospectively enrolled 423 patients with high clinical suspicion of RLS and divided them into the contrast transcranial Doppler (cTCD) group and synchronous multimode ultrasound group, in which both cTCD and contrast transthoracic echocardiography (cTTE) were performed during the same process of contrast-enhanced ultrasound imaging. The simultaneous test results were compared with those of cTCD alone.

**Results:**

The positive rates of grade II (22.0%:10.0%) and III (12.7%:10.8%) shunts and the total positive rate (82.1748%) in the synchronous multimode ultrasound group were higher than those in the cTCD alone group. Among patients with RLS grade I in the synchronous multimode ultrasound group, 23 had RLS grade I in cTCD but grade 0 in synchronous cTTE, whereas four had grade I in cTCD but grade 0 in synchronous cTTE. Among patients with RLS grade II in the synchronous multimode ultrasound group, 28 had RLS grade I in cTCD but grade II in synchronous cTTE. Among patients with RLS grade III in the synchronous multimode ultrasound group, four had RLS grade I in cTCD but grade III in synchronous cTTE. Synchronous multimode ultrasound had a sensitivity of 87.5% and specificity of 60.6% in the patent foramen ovale (PFO) diagnosis. Binary logistic regression analyses showed that age (odds ratio [OR] = 1.041) and risk of paradoxical embolism score ≥ 7 (OR = 7.798) were risk factors for stroke recurrence, whereas antiplatelets (OR = 0.590) and PFO closure with antiplatelets (OR = 0.109) were protective factors.

**Conclusion:**

Synchronous multimodal ultrasound significantly improves the detection rate and test efficiency, quantifies RLS more accurately, and reduces testing risks and medical costs. We conclude that synchronous multimodal ultrasound has significant potential for clinical applications.

## 1. Introduction

Patent foramen ovale (PFO) is a common congenital atrial septal defect in adults, with an incidence of 15–35% (1). PFO may lead to a right-to-left shunt (RLS), which is associated with migraine (2), cryptogenic stroke (3), and other conditions. RLS is an underappreciated cause of significant hypoxia, necessitating timely and accurate diagnosis to optimize management (4). The diagnosis of RLS is confirmed when microbubbles are observed in either the left atrium or ventricle on contrast transthoracic echocardiography (cTTE) and contrast transesophageal echocardiography (cTEE) or the middle cerebral arteries/vertebrobasilar artery on contrast transcranial Doppler (cTCD) after contrast injection. Reliable technology for detecting RLS/PFO is essential for the diagnosis and further treatment. Although cTCD, cTTE, and cTEE can be used to detect RLS/PFO (5), the most practical and cost-effective ultrasound modality remains debatable.

cTCD is a non-invasive, inexpensive, and easily repeatable screening tool for the detection of PFO. cTTE is a widespread initial screening tool for PFO because of its low cost, availability, and non-invasiveness (6). cTCD appears to be more sensitive than cTEE (7, 8); however, it is less specific than cTTE (9), cannot differentiate between cardiac and pulmonary shunts, and is easily affected by an inadequate acoustical temporal bone window. cTTE is disturbed by body position, subcutaneous fat in the thorax, and gas in the lungs (10). cTEE is regarded as the gold standard for the diagnosis of PFO (5); however, TEE is less sensitive than TTE in the diagnosis of RLS and underestimates shunt severity (11). The specificity is significantly improved when cTTE is combined with cTCD in detecting PFO (12). Some clinicians have used asynchronous cTCD and cTTE tests (multimode ultrasound) to screen for RLS (13). However, patients must undergo two contrast echocardiography procedures, which increase testing risks and costs.

In this study, we aimed to evaluate the effectiveness and feasibility of synchronous multimode ultrasound imaging to detect RLS by performing cTCD and cTTE during the same process of contrast-enhanced ultrasound.

## 2. Materials and methods

The study was approved by the Ethics Review Board of the First Affiliated Hospital of Shenzhen University (No. 20220413006). All the enrolled participants signed an informed consent form.

### 2.1. Study population

From January 2021 to September 2021, 250 consecutive patients admitted to the First Affiliated Hospital of Shenzhen University with clinical suspicion of RLS were prospectively enrolled. The 423 patients were divided into the cTCD and multimode ultrasound groups according to the decision of the physician in charge. Baseline demographic characteristics are shown in Table 1. In the multimode ultrasound group, 73 participants eventually completed cTEE or TEE.

**Table 1:** Baseline characteristics of the study population and the comparison of RLS grades in synchronous multimode ultrasound and cTCD groups.

Characteristics	Multimode ultrasound group	cTCD group	*t*/chi-squared value	*p*-value
Patients	173	250		
Age, years	49.2 ± 16.3	49.6 ± 15.6	−0.259	0.796
Men	102 (59.0%)	150 (60.0%)	0.046	0.454
Women	71 (41.0%)	100 (40.0%)		
Smoking	56 (32.4%)	80 (32.0%)	0.006	0.509
Hypertension	38 (22.0%)	55 (22.0%)	0.000	0.546
Diabetes mellitus	16 (9.2%)	22 (8.8%)	0.025	0.502
Cryptogenic stroke	109 (63.0%)	157 (62.8%)	0.002	0.524
Migraine	64 (37.0%)	93 (37.2%)	0.002	0.524
RLS grade 0	31 (17.9%)	63 (25.2%)	3.136	0.048
RLS grade I	82 (47.4%)	135 (54.0%)	1.783	0.108
RLS grade II	38 (22.0%)	25 (10.0%)	11.549	0.001
RLS grade III	22 (12.7%)	27 (10.8%)	0.367	0.324
Positive patients	142 (82.1%)	187 (74.8%)	3.136	0.048
Negative patients	31 (17.9%)	63 (25.2%)		

Data are expressed as number (%) or mean ± standard deviation (range).

cTCD, contrast transcranial Doppler; cTTE, contrast transthoracic echocardiography; RLS, right-to-left shunt; TCD, transcranial Doppler.

The diagnoses of cryptogenic stroke (14), hypertension (15), diabetes mellitus (16), and migraine (17) were confirmed based on the corresponding guidelines or diagnostic criteria. Patients with stroke are generally given secondary prevention drug treatment within 48 h of admission. Of the patients in this study, 10 out of 109 were stroke-related (the risk of paradoxical embolism [RoPE] score was ≥7) with large RLS and received surgical closure of PFO. The risk of paradoxical embolism was calculated based on the RoPE score, and surgical closure of PFO was proposed according to the Chinese expert consensus on preventive closure of patent foramen ovale (18). The average time from stroke onset to PFO occlusion was 50 days.

The inclusion criteria were as follows: age of 18–80 years, high clinical suspicion of RLS, voluntary participation, and good image quality in ultrasonic tests. The exclusion criteria were congenital heart diseases, such as atrial and ventricular septal defects, and the inability to perform the Valsalva maneuver (VM).

### 2.2. Activated saline contrast

Activated saline contrast consisted of 8 mL of saline solution, 1 mL of air, and 1 mL of patient’s blood. The resulting fluid was vigorously mixed with two 20 mL syringes using a three-way pipe at least 20 times. We administered an intravenous bolus injection of activated saline contrast into the antecubital vein (11) using an 18-gauge needle to allow the bolus to reach the right atrium. Activated saline contrast injection was performed once at rest and twice during the VM.

### 2.3. Methods of VM

The patients were trained to perform VM before the tests. The activated saline contrast was injected 5 s before the start of the VM. The patients were instructed to blow into a small soft plastic tube connector to a manometer. They were then required to maintain a minimum pressure of 40 mmHg for 5 s. The effective VM was identified by a peak Doppler flow velocity that decreased by at least 25% in the middle cerebral artery (11).

### 2.4. cTCD

**Figure 1:**
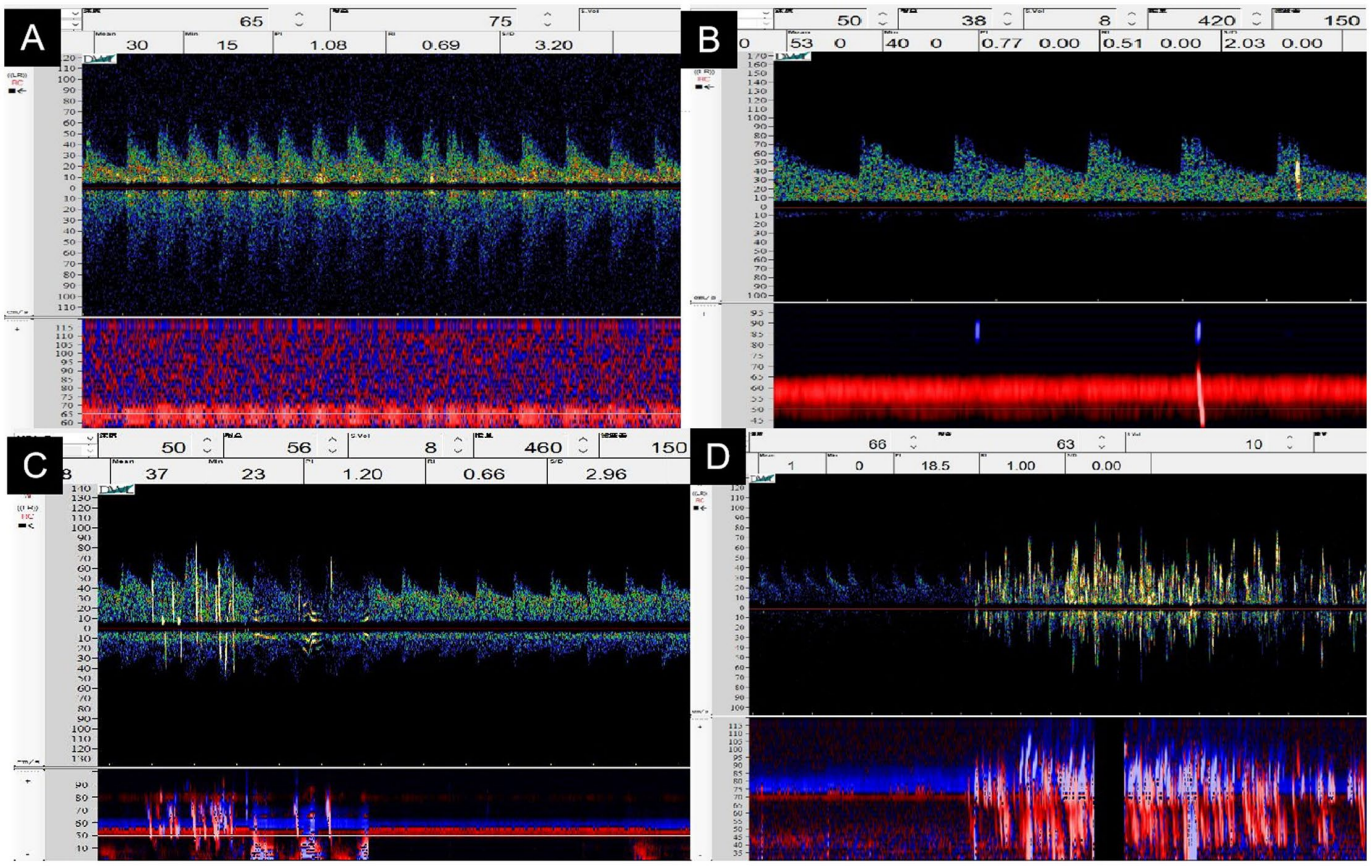
The grading classification of right-to-left shunts in contrast transcranial Doppler ultrasonography. **(A)** Grade 0_TCD_ = no microbubbles. **(B)** Grade I_TCD_ = 1–10 microbubbles (arrow). **(C)** Grade II_TCD_ = 10–30 microbubbles but no curtain (arrow). **(D)** Grade III_TCD_ = curtain-like microbubbles (arrow).

The cTCD test was performed using a TCD machine (DWL Doppler-Box; Germany) with a probe frequency of 2 MHz. For this test, participants were requested to lay in a comfortable left lateral position. We performed a single-channel TCD and double-depth monitoring, and the middle cerebral artery was observed through a temporal window. If the temporal window sound transmission was poor, a suboccipital window was selected to monitor the vertebral arteries. The first injection of activated saline contrast was conducted during rest and subsequently with the VM. The test was repeated after the first VM, and Doppler spectra were recorded for 25 s. We defined the cTCD outcome as positive for RLS when the TCD detected more than one microbubble within 25 s after the injection of activated saline (5). The test with the largest number of microbubbles was used to determine the RLS grade. The shunt grades (19) in the cTCD were as follows: grade 0 TCD (negative) = no microbubbles, grade I TCD = small shunt (1–10 microbubbles), grade II TCD = moderate shunt (10–30 microbubbles but no curtain), and grade III TCD = large shunt (more than 30 microbubbles, including the curtain pattern; Figure 1).

### 2.5. cTTE

**Figure 2:**
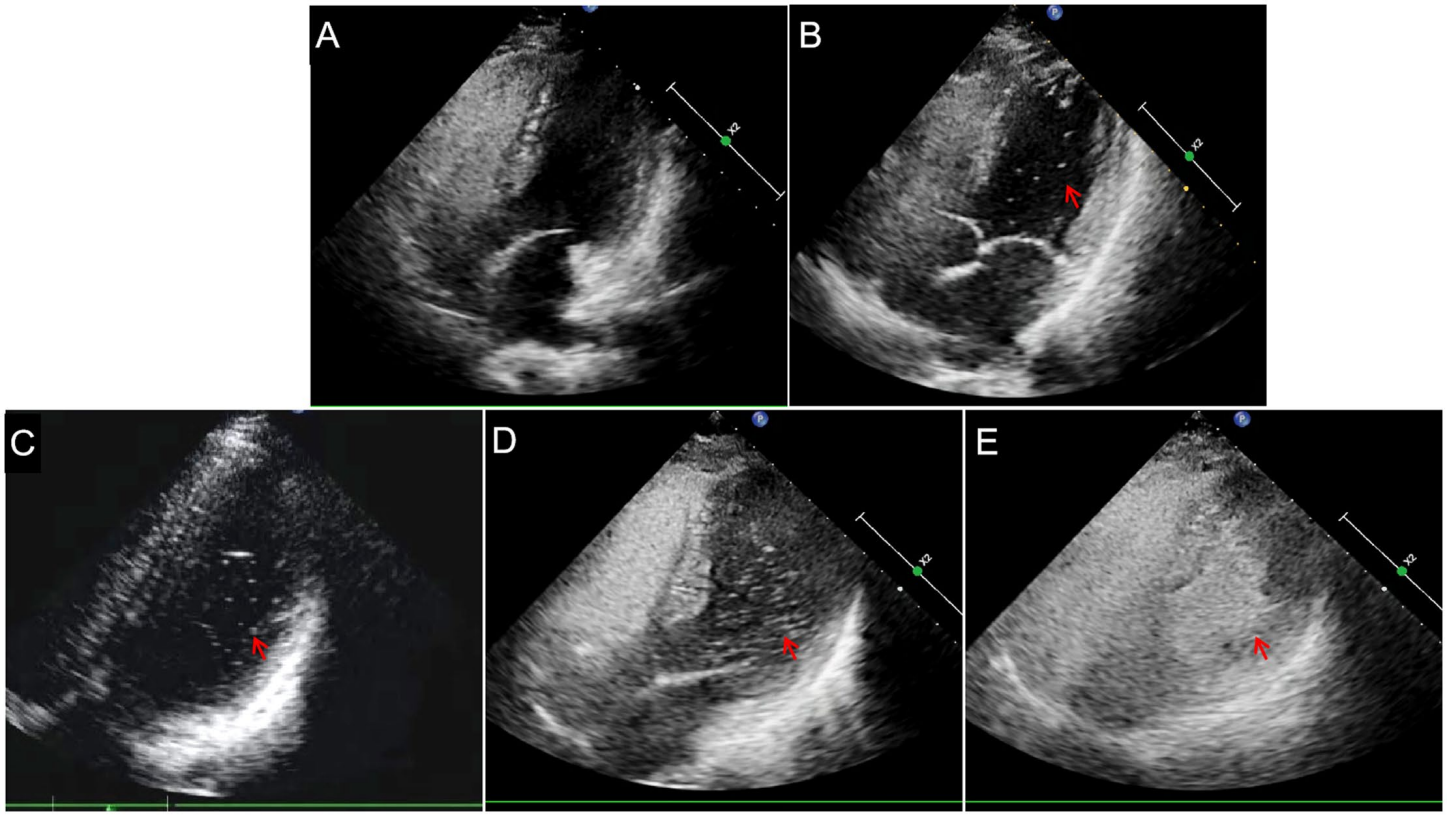
Semi-quantitative grading of right-to-left shunts in contrast transthoracic echocardiography. **(A)** Grade 0_TTE_ = negative. **(B)** Grade I_TTE_ = 1–10 microbubbles (arrow). **(C)** Grade II_TTE_ = 11–30 microbubbles (arrow). **(D)** Grade III_TTE_ = >30 microbubbles (arrow) or **(E)** the left atrium is nearly filled with microbubbles (arrow).

cTTE was performed with an EPIQ 7C Color Doppler Ultrasound instrument (Philips, the Netherlands), with an X5-1 probe and a frequency of 1.0–5.0 MHz. We focused the ultrasound probe on the atrial septum and observed whether an interruption of echo continuity in the atrial septum and color blood flow through the interrupted site of the atrial septum were present. We recorded continuously during the contrast injections using the apical four-chamber view. When the TTE detected microbubbles in the left atrium within 5–7 cardiac cycles, the cTTE results were considered positive. The test in which the most microbubbles appeared in the left atrium was considered for the final result. The degree of shunt severity in cTTE based on the detected microbubbles in the left atrium was quantified as follows (6): grade 0 TTE (negative) = no microbubbles, grade I TTE = 1–10 microbubbles, grade II TTE = 11–30 microbubbles, and grade III TTE = more than 30 microbubbles (the left atrium is nearly filled with microbubbles or left atrial opacity is present; Figure 2).

### 2.6. cTEE

cTEE was performed using a Philips EPIQ-7C instrument equipped with an X7-2t multiplane probe. All patients were fasting, and they were positioned in the left lateral decubitus position and received premedication with pharyngeal anesthesia using tetracaine. Blood pressure and electrocardiography findings were also monitored. Comprehensive two-dimensional color and spectral Doppler TEE were performed after the insertion of the probe. By observing the degree of involution of the foramen ovale valve under resting conditions, the presence of fissures between the septum primum and septum secundum, and the presence of color shunts observed by color Doppler (20), we confirmed the diagnosis of PFO when microbubbles (Supplementary Video 1) or color Doppler flow signals crossed from the foramen ovale (Supplementary Video 2). During the VM, the operator asked the patients to expand their abdomen and/or cough. An activated saline injection was performed posterior to the VM. We maintained continuous recording once during the basal condition and twice during the VM. The observations with the most microbubbles in the left atrium were regarded as the final results (13).

### 2.7. Synchronous multimode ultrasound imaging

Two experienced ultrasound technicians performed synchronous detection of cTTE and cTCD in 173 patients. Each technician was blinded to the results obtained by the other technician during the inspection process. Both tests were performed using the same activated saline, posture, and VM. A fixed examiner instructed the patients to perform the VM. The result of the cTTE or cTCD with the most microbubbles was regarded as the final RLS grade in synchronous testing.

### 2.8. Statistical analysis

All statistical analyses were performed using SPSS (version 22.0; SPSS Inc., Chicago, IL, United States). The measured data showed a normal distribution and were expressed as the mean ± standard deviation.

The independent-sample *t*-test was used for between-group comparisons for age. Categorical variables are summarized as counts, and the differences between the two groups were analyzed using the chi-squared test. Risk factors for stroke recurrence were analyzed using binary logistic regression analysis. Statistical significance threshold was set at *p* < 0.05.

## 3. Results

Table 1 presents the comparison of RLS manifestations between the synchronous multimode ultrasound and cTCD alone groups. The baseline data of the two groups were generally comparable (*p* > 0.05). However, the rate of grade II shunts (22.0%:10.0%) and total positive shunt rate (82.1%:74.8%) were significantly higher in the synchronous multimode ultrasound group than in the cTCD alone group (*p* = 0.001 and *p* = 0.048, respectively; Table 1).

Table 2 shows the comparison of RLS grades detected by cTCD with those detected by cTTE in patients from the synchronous multimode ultrasound group. Among the patients ultimately diagnosed with RLS grade I, 23 patients had RLS grade I in cTCD but grade 0 in synchronous cTTE, whereas four patients had RLS grade 0 in cTCD but grade I in synchronous cTTE. Among patients diagnosed with RLS grade II, 28 patients had RLS grade I in cTCD but RLS grade II in synchronous cTTE. Among patients diagnosed with RLS grade III, four patients had RLS grade I in cTCD but RLS grade III in synchronous cTTE.

**Table 2:** Comparison of RLS grades detected by cTCD with those detected by cTTE in the synchronous multimode ultrasound group.

RLS grade according to multimode ultrasound (cTCD + cTTE)	*N*	Shunt on cTCD	Shunt on cTTE
RLS grade 0, TN = 31	31	RLS grade 0	RLS grade 0
RLS grade I, TN = 82	55	RLS grade I	RLS grade I
	23	RLS grade I	RLS grade 0
	4	RLS grade 0	RLS grade I
RLS grade II, TN = 38	28	RLS grade I	RLS grade II
	4	RLS grade II	RLS grade II
	4	RLS grade II	RLS grade I
	1	RLS grade 0	RLS grade II
	1	RLS grade II	RLS grade 0
RLS grade III, TN = 22	15	RLS grade III	RLS grade III
	4	RLS grade I	RLS grade III
	3	RLS grade II	RLS grade III

cTCD, contrast transcranial Doppler; cTTE, contrast transthoracic echocardiography; N, number; RLS, right-to-left shunt; TN, total number.

Table 3 shows the effectiveness of synchronous multimode ultrasound for the diagnosis of RLS/PFO when compared to that afforded by cTEE as the gold standard. Synchronous multimode ultrasound had a sensitivity of 87.5% and a specificity of 60.6%.

**Table 3:** Diagnostic effectiveness of synchronous multimode ultrasound for the diagnosis of PFO-RLS using cTEE as the gold standard.

Synchronous multimode ultrasound (cTCD + cTTE)	PFO-RLS (diagnosed by cTEE)		Total
	Positive patients	Negative patients	
Positive patients	35	13	48
Negative patients	5	20	25
Total	40	33	73

cTCD, contrast transcranial Doppler; cTEE, contrast transesophageal echocardiography; cTTE, contrast transthoracic echocardiography; PFO, patent foramen ovale; RLS, right-to-left shunt.

Table 4 presents the comparison of RLS diagnoses in patients with cryptogenic stroke from the synchronous multimode ultrasound and cTCD alone groups. The total positivity rate (82.9%) was significantly higher as judged by the synchronous multimode ultrasound investigation than that reported by cTEE/TEE (61.4%, *p* = 0.005). RLS at all levels detected by multimode ultrasound was significantly different from that indicated by cTEE/TEE (*p* = 0.016).

**Table 4:** Comparison of effectiveness of TCD alone and multimode ultrasound in patients with cryptogenic stroke.

TN = 70	Multimode ultrasound group	cTCD group	cTEE or TEE group	*p*-value ^(Multimode ultrasound vs. TEE)^	*p*-value ^(cTCD vs. TEE)^
RLS grade 0	12 (17.1%)	17 (24.3%)	27 (38.6%)	0.016	0.321
RLS grade I	32 (45.7%)	38 (54.3%)	30 (42.9%)		
RLS grade II	16 (22.9%)	7 (10.0%)	7 (10.0%)		
RLS grade III	10 (14.3%)	8 (11.4%)	6 (8.6%)		
Positive patients	58 (82.9%)	53 (75.7%)	43 (61.4%)	0.005	0.069
Negative patients	12 (17.1%)	17 (24.3%)	27 (38.6%)		

cTCD, contrast transcranial Doppler; cTEE, contrast transesophageal echocardiography; RLS, right-to-left shunt; TEE, transesophageal echocardiography; TN, total number. The differences between the two groups were analyzed using the chi-squared test.

**Figure 3:**
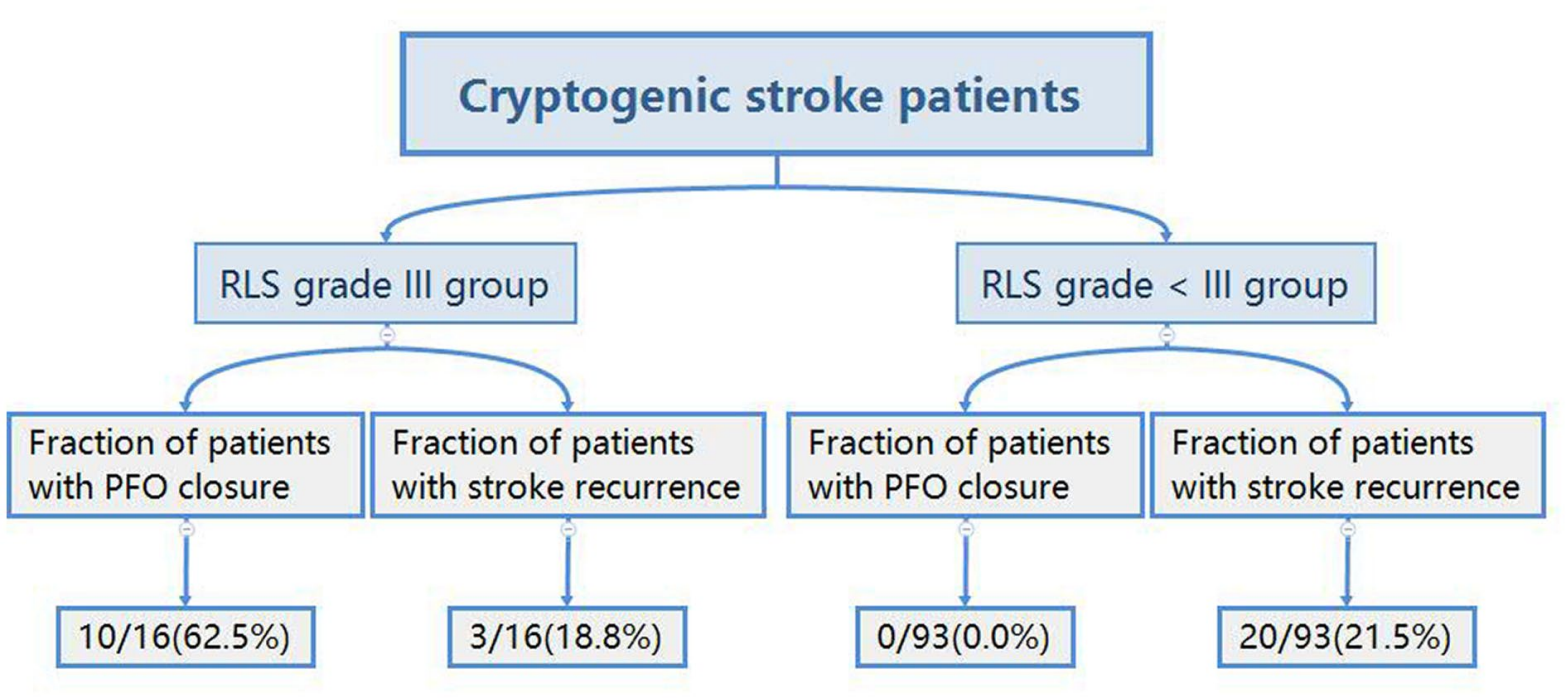
Flowchart of the state of patent foramen ovale closure and recurrence in patients with cryptogenic stroke. PFO, patent foramen ovale; RLS, right-to-left shunt.

Table 5 presents the comparison of clinical characteristics and stroke recurrence in patients with cryptogenic stroke that were diagnosed with RLS grade III and RLS grade < III. The rate of stroke recurrence was significantly different between these two groups (*p* = 0.013), and the RLS grade III group had a significantly higher number of patients with a RoPE score ≥ 7 (*p* = 0.001). The fractions of patients with cryptogenic stroke that had PFO closure and stroke recurrence are shown in Figure 3.

**Table 5:** Comparison of clinical characteristics between cryptogenic stroke patients with RLS grade III and those with RLS grade < III.

	RLS grade III	RLS grade < III	*p*-value
Patients	16	93	
Age, years	49.32 ± 15.97	46.35 ± 17.40	0.495
Men	9 (56.3%)	56 (60.2%)	0.486
Women	7 (43.8%)	37 (39.8%)	
Smoking	5 (31.3%)	29 (31.2%)	0.603
Hypertension	3 (18.8%)	19 (20.4%)	0.591
Diabetes mellitus	2 (12.5%)	8 (8.6%)	0.450
Antiplatelets	5 (31.3%)	88 (94.6%)	<0.001
Anticoagulation	1 (6.3%)	5 (5.4%)	0.727
PFO closure with antiplatelets	9 (56.3%)	0 (0.0%)	<0.001
PFO closure with anticoagulation	1 (6.3%)	0 (0.0%)	0.147
Patients with RoPE score ≥ 7	11 (68.8%)	28 (30.1%)	0.004
Patients with RoPE score < 7	5 (31.2%)	65 (69.9%)	
Stroke recurrence	3 (18.8%)	20 (21.5%)	0.551

RLS was diagnosed using multimode ultrasound, and the patients were followed up for 18 to 26 months after the first-ever stroke event. PFO, patent foramen ovale; RLS, right-to-left shunt; RoPE, risk of paradoxical embolism.

Table 6 presents the results of binary logistic regression analyses of stroke recurrence. Age (odds ratio [OR] = 1.041) and RoPE score ≥ 7 (OR = 7.798) were risk factors for stroke recurrence, whereas therapy with antiplatelet drugs (OR = 0.590) and PFO closure combined with antiplatelet therapy (OR = 0.109) were protective factors against stroke recurrence.

**Table 6:** Binary logistic regression analysis of stroke recurrence.

Selected variables	β	SE	Wald *χ*^2^	*p*-value	OR	95% CI	
						Lower bound	Upper bound
Age	0.041	0.017	5.804	0.016	1.041	1.008	1.077
Antiplatelets	−2.828	1.082	6.830	0.009	0.590	0.170	0.943
RoPE score ≥ 7	2.054	0.968	4.505	0.034	7.798	1.170	11.962
PFO closure with antiplatelets	−2.215	1.125	3.878	0.049	0.109	0.074	0.256

β, regression coefficient; CI, confidence interval; PFO, patent foramen ovale; OR, odds ratio; RoPE, risk of paradoxical embolism; SE, standard error.

We did not observe any complications during the synchronous multimode ultrasound.

## 4. Discussion

In this study, the rate of RLS detection by the simultaneous multimodal ultrasound (cTTE and cTCD) was higher than that by cTCD alone, especially for RLS grades II and III. We showed that synchronous multimode ultrasound imaging significantly improved the detection rate of RLS and provided a more accurate classification of this condition. These advantages can help to diagnose stroke or migraine and assist with treatment decision-making.

### 4.1. Advantages of synchronous multimode ultrasound imaging

Our study showed that TCD alone cannot reliably inform about the actual RLS grade and that using cTTE or cTCD for RLS/PFO screening is not completely interchangeable, as shown by the significant differences in the individual outcomes of simultaneous tests in the same patient (Table 2). Therefore, it is necessary to combine the results of both tests for the analysis. The synchronous test can significantly improve the total positive rate and detection rate of moderate/large RLS compared with the performance of the individual tests (Table 3).

Multimodal ultrasound imaging improved the sensitivity of RLS/PFO diagnosis for the following reasons: (1) Synchronous cTCD and cTTE combine the advantages of the two individual tests and avoid or reduce their shortcomings. This synergy increases the PFO detection rate. (2) The examiners need to count the number of microbubbles during cTTE. However, some microbubbles at certain levels, especially in small amounts, are not detected by the ultrasonic probe (21), and a single microbubble may be missed. (3) Obesity, pulmonary emphysema, other diseases, or VM affect the image quality of TTE (13), all of which can be avoided using cTCD. In cases of poor temporal bone windows or suspicious microbubble signals during cTCD (9), cTTE may be used to perform the test. (4) cTTE detects cardiac RLS directly in the heart, whereas cTCD detects only intracranial shunts, which are a subset of RLS (22). Therefore, the observed RLS values were not consistent. (5) Operators can mutually verify the examination results after the synchronous test of cTCD and cTTE, which helps gain additional experience.

Synchronous multimodal ultrasound can also improve the specificity of PFO diagnosis. cTCD neither displays an intracardiac structure nor identifies the source (e.g., pulmonary or cardiac) of RLS (13, 23). Furthermore, the atrial septum and intracardiac structures as well as the cardiac cycle in which the microbubbles appear can all be conveniently observed using cTTE, which helps determine whether there is a cardiogenic RLS. Most sonographers define a positive intracardiac RLS as comprising the passage of ≥1 microbubble into the left atrium within three cardiac cycles (10). It has been shown that the sensitivity and specificity for detecting PFO are higher within five cardiac cycles using cTTE (12). In addition, cTCD is a useful alternative for detecting PFO, especially for grades ≥ III (12). Therefore, a combination of cTCD and cTTE improves the specificity of PFO diagnosis.

### 4.2. Disadvantages of synchronous multimode ultrasound imaging

Multimode ultrasound imaging can yield false-positive or false-negative results. False-positive results may be due to the mischaracterization of intrapulmonary shunts as intracardiac shunts. False-negative results are typically due to respiratory-phasic variation, inadequate VM, or inadequate use of agitated saline (4). Multimode ultrasound can also increase the testing costs for patients with negative results compared with those associated with cTCD or cTTE alone.

### 4.3. Synchronous multimode ultrasound imaging compared with cTCD

In a previous study, the detection rates of RLS grade II for cTTE and cTCD were 9.2 and 8.9%, respectively, whereas those of RLS grade III were 15.0 and 12.1%, respectively (21), which is consistent with our study findings. The detection rates of RLS grades II and III in the synchronous multimode ultrasound test were higher than those afforded by cTCD alone (Table 1).

Diagnosis of PFO using TCD has high sensitivity (95%) and specificity (92%). Therefore, TCD should be recommended as the first choice for PFO screening (24). cTTE can detect the majority of RLSs with better specificity than that of cTCD (25). Many experts believe that both cTCD and cTTE should be used as initial screening tests for PFO (6). A previous study (5) showed that the specificity was significantly higher, and the misdiagnosis rate was lower when cTTE was combined with cTCD to diagnose PFO.

In our experience, synchronous multimode ultrasound imaging can save patients’ time, make the test process more efficient, reduce the risk of the test, workload, and medical cost, and increase patient compliance. Most hospitals use asynchronous testing (13), which consists of cTCD first, followed by cTTE, or vice versa. To complete these tests sequentially rather than simultaneously, patients need to undergo two contrast echocardiography procedures, which extend the duration of work, decreases test efficiency, and increases the cost. Moreover, cTCD and cTTE can be completed by different operators in different locations, which may impede joint discussion and communication. Synchronous cTCD and cTTE tests can address these shortcomings. This approach is non-invasive and can be simultaneously completed with only one intravenous contrast-enhanced ultrasound, improving patient compliance. Sonographers and neurologists should coordinate the test time and place and collaboratively assess the test results. A better multi-disciplinary cooperation is critically important because the shunt grading by cTCD and cTTE indicates the size of the PFO and the next course of treatment (26).

### 4.4. Reasons for the high number of patients positive for RLS

A previous study (27) reported that the rate of RLS detection by cTCD in patients with cryptogenic stroke was 58.5%. In our study, we found a higher number of patients positive for RLS. There could be several possible reasons for this discrepancy. First, the participants in our study were patients with cryptogenic stroke or migraine, were at high risk of RLS, and were of younger age. Second, we adopted a standardized and quantifiable VM procedure, and the participants were fully trained before the test. Third, the procedures carried out by our specially-trained nurses may have produced more effective contrast enhancement with activated saline injection. Fourth, when the right atrium was filled with microbubbles and became blurry during the cTTE test, the participants were asked to relax, which facilitated detection of RLS at that time point.

### 4.5. Analysis of cryptogenic stroke recurrence

Our study showed age and RoPE score ≥ 7 were risk factors for stroke recurrence. Age was shown to be a risk factor for ischemic stroke recurrence in patients with intracranial hemorrhage (28). In addition, older age is an independent predictor of atrial fibrillation after embolic stroke of undetermined source (ESUS) (29), and atrial fibrillation is a well-known risk factor for embolic stroke recurrence. The RoPE score positively correlates with the risk of stroke and is helpful in differentiating patients with ESUS and pathogenic PFO from those with incidental PFO (30).

Our study revealed that the use of antiplatelet treatment alone and PFO closure combined with antiplatelet treatment were protective factors against stroke recurrence. The subjects in this study received antiplatelet drugs more frequently than anticoagulants. Aspirin significantly reduced ischemic stroke recurrence (31). Anticoagulation therapy may reduce the risk of recurrent stroke more than antiplatelet therapy in patients with ESUS (32); however, there are also findings inconsistent with this notion (33). PFO closure in patients with cryptogenic stroke is superior to antithrombotic treatment in prevention of recurrence (34). PFO closure can be considered for the prevention of recurrent cryptogenic stroke in patients aged ≤60 years after a thorough evaluation, and PFO closure with medical therapy is more cost-effective than medical therapy alone (35).

### 4.6. Limitations

The limitation of this study is that although we included a large number of patients, it was performed at a single center, and the results are only applicable to the selected population group. Larger multicenter studies evaluating more patients are needed to confirm our findings. Cooperating with cardiologists can help improve the methods and techniques for PFO detection.

## 5. Conclusion

We showed that screening for RLS/PFO using cTTE or cTCD was not completely interchangeable, and both cTCD and cTTE are necessary and complementary for detecting RLS/PFO. Synchronous multimode ultrasound (cTCD and cTTE) can significantly improve the detection rate and quantification of RLS, increase the test process efficiency, and reduce facility risks and medical costs. This test requires multi-disciplinary cooperation. We conclude that synchronous multimode ultrasound has great potential for clinical applications and deserves further investigation.

## Data availability statement

The original contributions presented in the study are included in the article/Supplementary material, further inquiries can be directed to the corresponding author.

## Ethics statement

The studies involving human participants were reviewed and approved by the Ethics Review Board of the First Affiliated Hospital of Shenzhen University (No. 20220413006). The patients/participants provided their written informed consent to participate in this study.

## Author contributions

LC and HX conceived the study, critically revised the manuscript, and obtained funding. QY conceived the study and wrote the first draft. DZ and SR provided constructive discussions and translated the manuscript. XZ, YZ, SH, and JW collected the data. WQ provided constructive discussions. All authors have read and approved the final manuscript.

## Funding

This study was supported by the Shenzhen Second People’s Hospital Clinical Research Fund of the Guangdong Province High-level Hospital Construction Project (nos. 20223357021 and 20213357016), Guangdong Natural Science Funds (no. 2020B1515120061), and Department of Shenzhen Local Science and Technology Development (no. 2021Szvup052).

## Conflict of interest

The authors declare that the research was conducted in the absence of any commercial or financial relationships that could be construed as a potential conflict of interest.

## Publisher’s note

All claims expressed in this article are solely those of the authors and do not necessarily represent those of their affiliated organizations, or those of the publisher, the editors and the reviewers. Any product that may be evaluated in this article, or claim that may be made by its manufacturer, is not guaranteed or endorsed by the publisher.

## Supplementary material

SUPPLEMENTARY VIDEOS S1, S2 The presence of a patent foramen ovale was confirmed when microbubbles (Video 1) or color Doppler flow signals (Video 2) crossed the foramen ovale.

## Abbreviations

cTCD: contrast transcranial Doppler
cTTE: contrast transthoracic echocardiography
ESUS: embolic stroke of undetermined source
OR: odds ratio
PFO: patent foramen ovale
RLS: Right-to-left shunt
RoPE: risk of paradoxical embolism
VM: Valsalva maneuver
